# Case report: Progressive organ-threatening IgG4-related disease despite conventional immunosuppression: lessons from a 5-year longitudinal follow-up

**DOI:** 10.3389/fimmu.2026.1975295

**Published:** 2026-10-28

**Authors:** Xinyue Zhang, Xiaoyue Deng, Jiaxuan Li, Yanqiu Zhang, Shixiong Wei, Xiaofeng Zeng

**Affiliations:** 1 Department of Rheumatology and Clinical Immunology, Peking Union Medical College Hospital, Chinese Academy of Medical Sciences, Peking Union Medical College, Beijing, China; 2 National Clinical Research Center for Dermatologic and Immunologic Diseases (NCRC-DID), Ministry of Science & Technology, Beijing, China; 3 Key Laboratory of Rheumatology and Clinical Immunology, Ministry of Education, Beijing, China; 4 State Key Laboratory of Complex Severe and Rare Diseases, Peking Union Medical College Hospital, Beijing, China; 5 Department of Endocrinology, Rheumatology and Immunology, Chengde Central Hospital, Chengde, Hebei, China; 6 Department of Rheumatology and Immunology, Union Hospital, Tongji Medical College, Huazhong University of Science and Technology, Wuhan, China

**Keywords:** csDMARDs, IgG4-related disease, IgG4-related hypophysitis, long-term follow-up, serum IgG4

## Abstract

**Objective:**

To describe the 5-year clinical course of a patient who progressed from IgG4-related dacryoadenitis and sialadenitis (IgG4-DS) to IgG4-related hypophysitis (IgG4-RH) and retroperitoneal fibrosis, evaluating the clinical course and treatment response to conventional immunosuppressive therapy through longitudinal clinical, serological, endocrine, and magnetic resonance imaging (MRI) assessments.

**Methods:**

Clinical records were retrospectively reviewed, focusing on disease progression after treatment discontinuation, responses to sequential conventional synthetic disease-modifying antirheumatic drugs (csDMARDs), and long-term pituitary endocrine outcomes.

**Results:**

During 5 years of follow-up, a 44-year-old man with IgG4-DS developed partial anterior hypopituitarism, central diabetes insipidus, and retroperitoneal fibrosis following two episodes of unplanned treatment discontinuation. Serial MRI demonstrated progressive pituitary stalk thickening accompanied by heterogeneous enhancement. The maximum stalk diameter was 4.0 mm at diagnosis (May 2023), decreased to 3.6 mm during maintenance therapy (September 2024), increased to 8.5 mm at relapse (March 2025), and decreased to 7.0 mm after treatment modification (November 2025). Glucocorticoids combined with sequential csDMARDs controlled acute inflammation but failed to prevent permanent gonadal and posterior pituitary dysfunction. Serum IgG4 reached a peak of 38,100 mg/L, and its re-elevation preceded both clinical and radiological relapse. At the last assessment in January 2026, serum IgG4 remained elevated at 4,594 mg/L, with persistent hypogonadism and central diabetes insipidus.

**Conclusions:**

Disease progression occurred mainly during treatment interruption, while residual activity persisted despite conventional csDMARDs. The relative contributions of treatment non-adherence and limited treatment efficacy remain uncertain. Combined serum IgG4 and MRI monitoring may aid early detection of relapse, but this requires validation in larger cohorts.

## 1 Introduction

IgG4-related disease (IgG4-RD) is a systemic fibro-inflammatory disorder characterized by elevated serum IgG4 levels, IgG4-positive plasma cell infiltration, and fibrosis in affected organs. The most commonly involved organs include salivary glands, pancreas, biliary tract, and retroperitoneum (1).Although IgG4-RD is characterized by IgG4 elevation, its pathogenesis is not mediated by IgG4 alone. IgG4 has limited complement- and Fc-mediated effector activity and is therefore unlikely to be directly pathogenic (2). B cells, circulating plasmablasts, and clonally expanded CD4+ cytotoxic T cells are instead considered key drivers of inflammation and fibrosis (3, 4). Elevation of total IgG and other subclasses, particularly IgG1, is common, while IgG1/IgG3-mediated complement activation may contribute to some phenotypes; moreover, serum IgG4 does not consistently reflect disease activity (5). Thus, non-IgG4 subclasses, particularly IgG1, may also contribute to tissue injury.

IgG4-related hypophysitis (IgG4-RH) is a rare manifestation of IgG4-RD, accounting for fewer than 4% of reported cases (5). Although growing clinical recognition of IgG4-related hypophysitis has improved its diagnostic detection rate, longitudinal data, particularly those documenting the dynamic evolution of pituitary imaging abnormalities, remain limited. IgG4-RH typically presents with headache, visual impairment, anterior pituitary dysfunction, and central diabetes insipidus. Characteristic MRI findings include pituitary stalk thickening, diffuse pituitary enlargement, and loss of the posterior pituitary bright spot (6). Although glucocorticoids remain the first-line therapy, relapse after tapering or discontinuation is frequent, and optimal long-term management remains uncertain (7).

Here, we report a case of IgG4-RH with 5 years of longitudinal clinical, serological, and pituitary MRI follow-up, illustrating disease evolution from isolated IgG4-related dacryoadenitis and sialadenitis (IgG4-DS) to multi-system involvement and progressive pituitary dysfunction.

## 2 Case presentation

### 2.1 Initial presentation and treatment at the referring hospital

A 44-year-old man presented to a local secondary hospital in February 2020 with a 9-month history of painful right submandibular gland swelling, where he was started on methylprednisolone (MP) 24 mg once daily and leflunomide (LEF) 20 mg once daily. He was referred to our center in November 2020 for diagnostic clarification. Laboratory examination revealed elevated serum IgG4 (6,840 mg/L) and IgE (1,302 IU/mL), and IgG4-DS was diagnosed. Treatment was continued with MP 4 mg once daily and LEF 20 mg once daily. He attended regular outpatient follow-up at our center at 3–6 month intervals from June 2021 onwards.

His medical history included chronic hepatitis B virus (HBV) infection diagnosed more than 10 years previously (HBsAg-positive, anti-HBe-positive, anti-HBc-positive), for which he had been followed at a local infectious disease hospital and registered in the national infectious disease registry, and was receiving tenofovir disoproxil fumarate 300 mg once daily. His liver function tests remained normal throughout the disease course and HBV serological and virological markers remained stable.

### 2.2 First treatment interruption and first relapse

In September 2021, after a period of clinical improvement, the patient discontinued all medications on his own initiative, based on the erroneous belief that he had recovered. Three months later, bilateral submandibular and right lacrimal gland enlargement recurred, accompanied by a marked increase in serum IgG4 (38,100 mg/L), with an erythrocyte sedimentation rate (ESR) of 16 mm/h and C-reactive protein (CRP) of 1.64 mg/L. Prednisone combined with cyclophosphamide induced remission, with subsequent decline of serum IgG4. Maintenance therapy was switched to mycophenolate mofetil after cyclophosphamide induction, and glucocorticoids were gradually tapered.

### 2.3 Second treatment interruption and onset of pituitary involvement

In July 2022 the patient again discontinued all medications on his own initiative after feeling clinically improved. Two months later, non-contrast cranial CT demonstrated only a small hyperdense focus in the suprasellar cistern, with no corresponding abnormality on the MRI performed at that time. Clinical symptoms, including fatigue, cold intolerance, reduced libido, weight loss, polydipsia, and nocturia, appeared approximately six months after discontinuation. A repeat pituitary MRI in January 2023 showed sellar enlargement with a 15×7×9 mm intrasellar mass exhibiting a waist sign, and contrast-enhanced imaging revealed an 8×7×6 mm enhancing suprasellar lesion inseparable from the pituitary stalk, with upward displacement of the optic chiasm. Abdominal imaging simultaneously demonstrated retroperitoneal fibrosis. Glucocorticoid therapy was reinitiated.

### 2.4 Admission, diagnosis and diagnostic certainty

At admission in May 2023, serum IgG4 remained markedly elevated (19,407 mg/L), with prolactin (PRL) at 10.9 ng/mL, free T4 (FT4) 0.82 ng/dL with thyroid-stimulating hormone (TSH) 2.461 µIU/mL, adrenocorticotropic hormone (ACTH) 49.0 pg/mL with morning cortisol 11.6 µg/dL, and insulin-like growth factor 1 (IGF-1) was low at 46 ng/mL. MRI showed a maximum pituitary stalk diameter of 4.0 mm (**Figures 1A–C**). Based on the clinical, serological and imaging findings, IgG4-RH with partial anterior hypopituitarism and partial central DI was diagnosed. Histopathological confirmation was unavailable because the patient declined pituitary and other organ biopsies. Thus, diagnostic certainty was limited, and alternative inflammatory or neoplastic etiologies could not be completely excluded. Treatment was initiated on 5 June 2023 with prednisone 60 mg/day and cyclophosphamide (CTX) 100 mg/day (**Supplementary Table S1**), resulting in a rapid decline in serum IgG4, improvement of retroperitoneal fibrosis (RPF), and stable pituitary stalk thickness (3.6 mm on MRI in September 2024). A gonadotrophin-releasing hormone (GnRH) stimulation testing with subcutaneous triptorelin (0.1 mg) on 23 October 2023 showed increases in luteinizing hormone (LH) (2.83 to 13.95 mIU/mL) and FSH (7.62 to 14.29 mIU/mL)at 60 min, indicating partially preserved gonadotroph reserve at that time.

**Figure 1:**
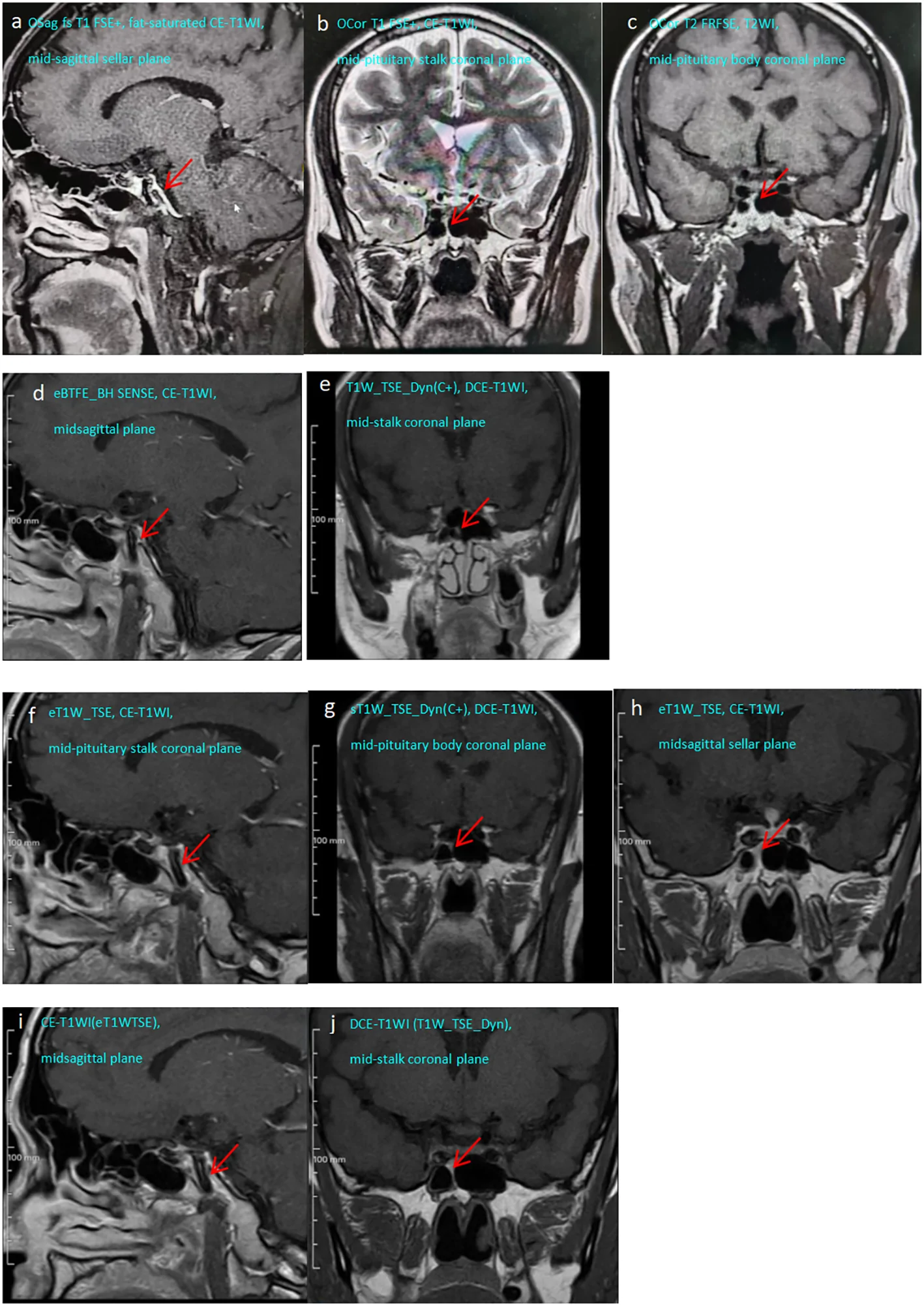
Longitudinal changes in pituitary MRI during follow-up. Maximum pituitary stalk diameter was 4.0 mm at diagnosis (**(A–C)**, May 2023), 3.6 mm during maintenance therapy (**(D, E)**, September 2024), 8.5 mm at relapse (**(F–H)**, March 2025) and 7.0 mm after treatment modification (**(I, J)**, November 2025). Each subpanel is labeled with its sequence and imaging plane (for example, fat-saturated contrast-enhanced T1-weighted imaging, mid-sagittal sellar plane), and arrows indicate the abnormal pituitary stalk. Contrast-enhanced images were acquired after intravenous administration of gadopentetate dimeglumine (Gd-DTPA) in all examinations.

### 2.5 Maintenance phase, serological rise and second relapse

After cyclophosphamide induction, maintenance therapy was changed to methotrexate with gradual glucocorticoid tapering. Persistent hypogonadism required testosterone replacement therapy (**Supplementary Table S1**). Pituitary MRI remained stable during follow-up, while methylprednisolone was tapered to 1.25mg/day. In November 2024, the patient remained clinically and radiologically stable (ESR 2 mm/h, CRP 1.25 mg/L), although serum IgG4 increased from a nadir of 2,535 to 6,807 mg/L. Prednisone was therefore increased to 5 mg/day and maintained thereafter, with no treatment interruption through the last follow-up.

MRI at the referring hospital in March 2025 showed recurrent nodular pituitary stalk thickening to 8.5 mm (**Figures 1F–H**). In April 2025, this was considered a clinical and radiological relapse. Laboratory evaluation revealed serum IgG4 was 7,447 mg/L, with PRL 34.7 ng/mL, FSH 0.41 IU/L, LH <0.2 IU/L, FT4 0.73 ng/dL, TSH 2.355 µIU/mL, ACTH 30.6 pg/mL, cortisol 7.7 µg/dL, and IGF-1–54 ng/mL. Methotrexate (MTX) was discontinued and mycophenolate mofetil (MMF) 1.0 g twice daily initiated on 8 April 2025, resulting in clinical improvement and partial regression of stalk thickening to 7.0 mm (**Figures 1I, J**) on MRI in November 2025. B-cell-targeted biologic therapy was recommended but declined for financial reasons, no B-cell depletion therapy had been administered by manuscript submission.

### 2.6 Last follow-up

At the last follow-up in January 2026, serum IgG4 decreased to 4,594 mg/L. Persistent hypogonadism and posterior pituitary dysfunction remained, whereas thyroid and adrenal axes were preserved. The patient remained clinically stable on hormone replacement therapy and MMF. The major clinical milestones are summarized in **Figure 2**.

**Figure 2:**
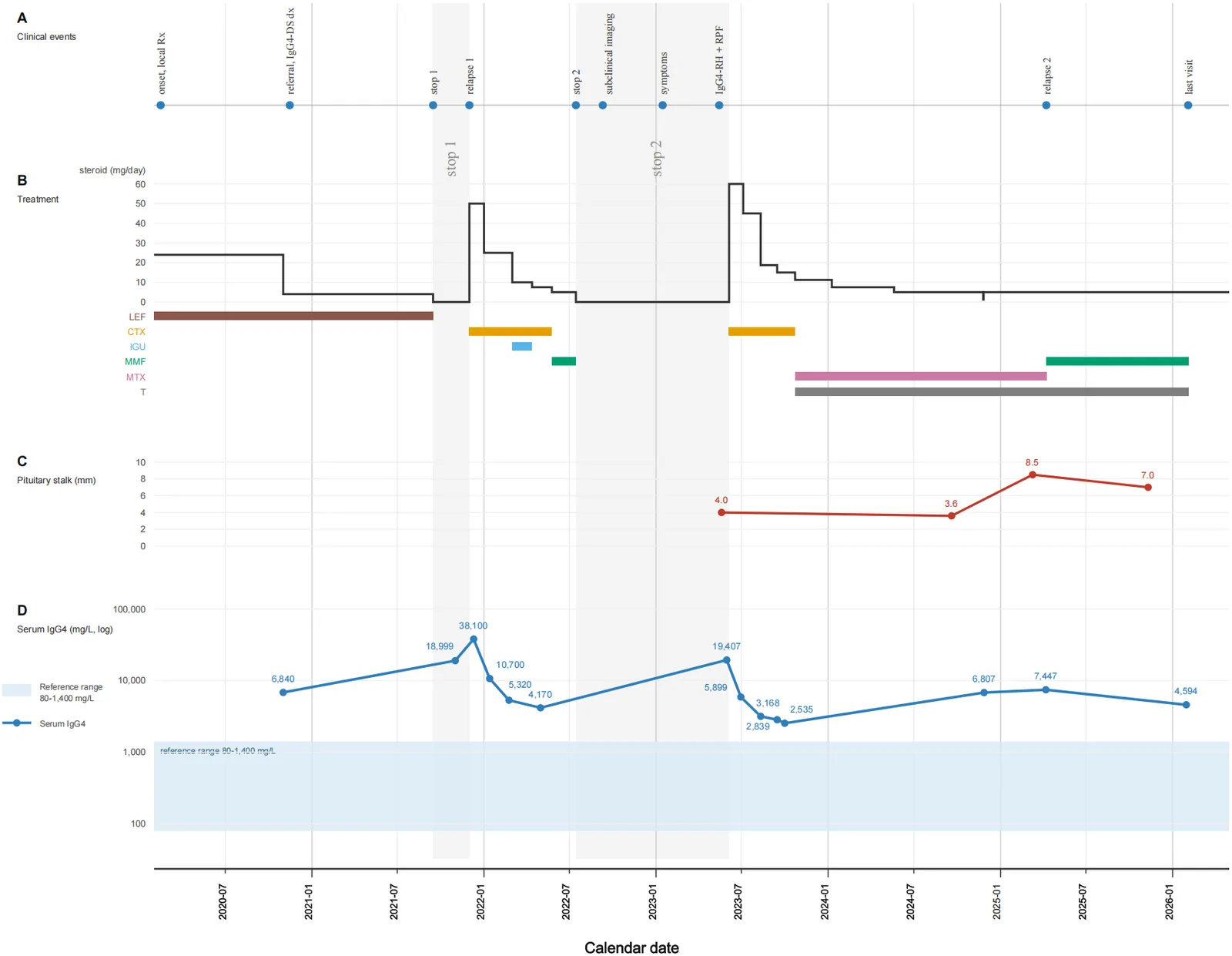
Integrated chronological timeline of the disease course. This figure presents an integrated chronological timeline covering the entire 5-year follow-up period of the patient, including: **(A)** Clinical and organ events. **(B)** Treatment exposure: the upper panel shows the glucocorticoid dose (mg/day, step plot); the lower panel shows the duration of each immunosuppressive agent, with repeated courses of the same drug shown on one row. **(C)** Maximum pituitary stalk diameter on contrast-enhanced MRI (gadopentetate dimeglumine; red). **(D)** Serum IgG4 concentration on a logarithmic scale (blue); the shaded band indicates the reference range (80–1,400 mg/L). All panels share one calendar-proportional x axis, and gray vertical bands indicate the two documented periods of complete treatment withdrawal (September-December 2021 and July 2022-June 2023). CTX, cyclophosphamide; IGU, iguratimod; LEF, leflunomide; MMF, mycophenolate mofetil; MTX, methotrexate; RPF, retroperitoneal fibrosis; T, testosterone.

### 2.7 Adverse events

Iguratimod was stopped in April 2022 because of gastric discomfort. The cumulative cyclophosphamide dose was monitored prospectively and the drug was replaced by MMF in May 2022 and by MTX in October 2023 once the cumulative dose was reached. No other adverse events attributable to glucocorticoids, leflunomide, MMF, MTX or tenofovir were documented during follow-up.

## 3 Methods

Study design and data sources: This retrospective case report used data from the electronic medical records of Peking Union Medical College Hospital, including inpatient and outpatient records, laboratory results, and radiological reports and images. The patient was followed up at our center for 5 years and 3 months (November 2020–February 2026).

Definitions: Clinical remission: Resolution of disease-attributable symptoms, with stable or improved organ function and no radiological progression.

Clinical relapse: Recurrence or worsening of disease-attributable symptoms, accompanied by elevation of serum IgG4 or progressive endocrine dysfunction, requiring treatment intensification.

Radiological remission: Reduction or stabilization of pituitary stalk thickness and lesion size on follow-up contrast-enhanced MRI.

Radiological progression: Increase in pituitary stalk thickness or lesion size, or appearance of new structural abnormalities on MRI.

Follow-up: Clinical and laboratory assessments were performed every 3–6 months, with pituitary MRI approximately every 6–12 months or as clinically indicated. Endocrine function was assessed at each visit from May 2023 onward.

Laboratory assays: Serum IgG4, IgG1, IgG2, IgG3, and total IgG were measured by nephelometry using the BN II System (Siemens Healthineers, Germany).Reference intervals were 80–1,400 mg/L for IgG4, 4,900–11,400 mg/L for IgG1, 1,500–6,400 mg/L for IgG2, 80–1,400 mg/L for IgG3, and 7.00–17.00 g/L for total IgG (**Supplementary Table S2**).

Endocrine hormones including IGF-1, FSH, LH, total testosterone, FT4, TSH, prolactin, and morning cortisol were measured by chemiluminescent immunoassay using the ADVIA Centaur XP system (Siemens Healthineers, Germany), with standard institutional reference ranges. Endocrine assessments were performed in the same laboratories, with reference intervals provided in **Supplementary Table S3**.

Imaging: Pituitary MRI was performed at our institution or the referring hospital using routine clinical protocols. Contrast-enhanced T1-weighted and T2-weighted sequences were obtained in sagittal and coronal planes, with sequence details provided in **Supplementary Table S4**. Gadopentetate dimeglumine (0.1 mmol/kg) was used for contrast-enhanced examinations. Pituitary stalk diameter was defined as the maximum diameter measured on contrast-enhanced T1-weighted images in the mid-sagittal and mid-stalk coronal planes and was obtained from the clinical radiology reports. No blinded independent re-measurement was performed. Because MRI examinations were obtained on different scanners and protocols, small longitudinal differences in stalk diameter were interpreted cautiously.

## 4 Discussion

### 4.1 Clinical characteristics and diagnostic certainty in the absence of a histological gold standard

A notable feature of this case was the selective impairment of pituitary hormonal axes. Persistent hyperprolactinemia accompanied by progressive gonadotropin deficiency suggested the coexistence of pituitary stalk effect and anterior pituitary involvement (8). In this patient, marked stalk thickening, elevated prolactin levels, and persistent gonadal axis dysfunction indicated combined stalk-mediated and inflammatory injury of the pituitary gland. The partial recovery of IGF-1 after immunosuppressive therapy suggested residual growth hormone axis function, whereas gonadal axis impairment appeared persistent. These findings highlight the heterogeneous vulnerability of pituitary hormonal axes to IgG4-related inflammatory and fibrotic damage (8).

Pituitary biopsy remains the diagnostic gold standard for IgG4-RH but is rarely performed because of the potential risk of irreversible pituitary dysfunction (9). As no biopsy of any other involved organs were performed in this patient, the diagnosis was highly supported by multisystem involvement, characteristic pituitary MRI findings, markedly elevated serum IgG4 levels, and a favorable response to immunosuppressive therapy, fulfilling the 2019 ACR/EULAR classification criteria for IgG4-RD and the diagnostic criteria proposed by Leporati et al (9, 10). Nevertheless, the absence of histopathological confirmation limits diagnostic certainty, and alternative inflammatory or neoplastic disorders cannot be fully excluded.

As pituitary and other organ biopsies were declined, alternative causes of pituitary/stalk disease were systematically evaluated. Investigations were negative or normal for ANCA (PR3 and MPO), serum calcium, angiotensin-converting enzyme, chest CT findings, tuberculosis, syphilis, fungal infection, HIV, EBV and CMV serology, serum alpha-fetoprotein and β-human chorionic gonadotrophin, and tumor markers. Lactate dehydrogenase was mildly elevated in 2023–2024 (255–361 U/L; reference 0–250 U/L) but normalized thereafter (214 U/L at last follow-up). Whole-body-18F-FDG PET/CT, long-bone imaging, and cerebrospinal fluid analysis were not performed, limiting the exclusion of alternative diagnoses. **Table 1** summarizes the evaluation of the major differential diagnoses.

**Table 1:** Differential diagnosis of pituitary and pituitary-stalk lesions in this patient.

Differential diagnosis	Typical features supporting the diagnosis	Findings in this patient	Judgment and basis	References
IgG4-RH (index diagnosis)	Multisystem IgG4-RD; stalk thickening or pituitary enlargement; elevated serum IgG4; steroid responsiveness;	Salivary, lacrimal, retroperitoneal and pituitary involvement; IgG4 19,407 mg/L at admission (peak 38,100); stalk 4.0 mm at diagnosis, 8.5 mm at relapse	Highly probable; no histopathology (patient declined biopsy)	(9, 10)
Lymphocytic hypophysitis	Predominant pituitary/stalk involvement; loss of posterior bright spot; steroid-responsive	Overlapping features; IgG4-RH was regarded as a subtype	Cannot be separated without histology; managed within the same spectrum	(11)
Granulomatous hypophysitis	Granulomatous infiltration; often panhypopituitarism	No supporting evidence; no biopsy	Cannot be excluded histologically	(12)
Granulomatosis with polyangiitis	Sino-nasal, pulmonary or renal involvement; PR3/MPO-ANCA positive	No sino-nasal, pulmonary or renal involvement; ANCA (PR3, MPO) negative	Unlikely	(13)
Sarcoidosis	Bilateral hilar lymphadenopathy; elevated ACE; hypercalcaemia	Chest CT: no features of sarcoidosis; serum calcium and serum angiotensin-converting enzyme normal	Unlikely	(14)
Erdheim–Chester disease	Long-bone osteosclerosis; retroperitoneal fibrosis; stalk/pituitary involvement; BRAF V600E	Retroperitoneal fibrosis present (shared feature); long-bone imaging and BRAF testing not performed	Cannot be fully excluded; stated as a limitation	(15)
Langerhans cell histiocytosis	Central DI; stalk thickening; lytic bone lesions common in the pediatric population	Skeletal imaging not performed Patient is a mid-age man	Unlikely	(16)
Infectious hypophysitis (tuberculosis, syphilis, fungal, HIV, EBV, CMV)	Fever or systemic features; risk factors	Tuberculosis, syphilis and fungal screening negative; HIV, EBV and CMV negative; liver function normal; HBV stable	Unlikely	(17)
Pituitary adenoma	Usually intrasellar; endocrine hypersecretion	Mass with waist sign; PRL elevated only at relapse (34.7 ng/mL) without an adenoma-typical pattern; no histology	Unlikely, but not histologically excluded	(18)
Rathke cleft cyst or pituitary apoplexy	Non-enhancing T1-hyperintense cyst; acute headache or visual loss	Transient non-contrast versus contrast discrepancy that later became homogeneous enhancement	Considered unlikely	(19, 20)
Germ cell tumor	Young patients; central DI as dominant feature; elevated AFP or beta-hCG (serum or CSF)	Serum AFP and beta-hCG normal; CSF not examined	Unlikely	(21)
Primary CNS lymphoma, metastasis or other malignancy	Contrast-enhancing mass; systemic malignancy; transient steroid response	Serum LDH only mildly elevated (255–361 U/L, 2023–2024); tumor marker panel normal; no PET/CT	Unlikely, although whole-body imaging was not performed	(18)

### 4.2 Longitudinal MRI evolution of IgG4-RH

Pituitary MRI is essential for the diagnosis and longitudinal assessment of IgG4-RH. Typical imaging features include diffuse or nodular pituitary enlargement, pituitary stalk thickening, loss of the posterior pituitary bright spot on T1-weighted imaging, and homogeneous intense enhancement, reflecting lymphoplasmacytic infiltration, IgG4-positive plasma cell accumulation, and microvascular proliferation (22). This case provides a rare longitudinal observation of MRI changes throughout the course of IgG4-RH.

Initially, MRI revealed only a small suprasellar nodular abnormality without clinical symptoms. Four months later (January 2023), the lesion had progressed to sellar enlargement with a 15×7×9 mm intrasellar mass exhibiting a waist sign and an 8×7×6 mm enhancing suprasellar lesion inseparable from the pituitary stalk, with upward displacement of the optic chiasm. During acute exacerbation, an unusual discrepancy between non-enhanced and contrast-enhanced MRI was observed, with a 15-mm sellar lesion identified on non-contrast imaging but only an approximately 8-mm uniformly enhancing suprasellar lesion after contrast administration. Such a pattern has rarely been reported in IgG4-RH. Notably, the atypical enhancement was observed only during acute exacerbation, whereas subsequent MRI consistently showed homogeneous enhancement. Given that structural lesions such as Rathke’s cleft cysts, ischemic lesions, and necrotic changes rarely evolve into uniformly enhancing lesions (19, 20), we hypothesize that this transient discrepancy may have reflected reversible inflammation rather than irreversible tissue destruction, although this interpretation remains speculative.

Following immunosuppressive therapy, MRI demonstrated regression of the enlarged lesion but persistent mild stalk thickening. Meanwhile, irreversible posterior pituitary dysfunction developed, accompanied by loss of the posterior pituitary bright spot. Imaging remained relatively stable during maintenance therapy; however, relapse after glucocorticoid tapering was associated with recurrent nodular stalk thickening (maximum diameter, 8.5 mm), suggesting renewed inflammatory activity and chronic structural remodeling. Serial measurements of the maximum pituitary stalk diameter were 4.0 mm at diagnosis (May 2023) and 3.6 mm during maintenance therapy (September 2024); the diameter subsequently increased to 8.5 mm at relapse (March 2025) and partially regressed to 7.0 mm after treatment modification (November 2025).

Overall, this case demonstrates the longitudinal MRI evolution of IgG4-RH, ranging from early subclinical abnormalities and acute inflammatory changes to post-treatment regression and chronic remodeling. The observation that MRI abnormalities preceded clinical manifestations (imaging change in September 2022, symptoms in January 2023) highlights the potential value of serial pituitary MRI for early detection and disease monitoring, although further validation is required. Longitudinal MRI findings are presented in **Figure 1**.

### 4.3 Serum IgG4 dynamics and the contribution of other IgG subclasses

Serum IgG4 is the widely used biomarker for assessing IgG4-RD activity (23). In this case, serum IgG4 levels closely paralleled treatment exposure, rising from 6840 mg/L at diagnosis to 18,999 mg/L 3 months after the first treatment interruption and peaking at 38,100 mg/L during relapse. It decreased to 4,170 mg/L with prednisone and cyclophosphamide, rose again to 19,407 mg/L after the second treatment interruption and onset of pituitary involvement, and reached a nadir of 2,535 mg/L during cyclophosphamide induction. At relapse in April 2025, serum IgG4 increased to 7,447 mg/L and subsequently decreased to 4,594 mg/L at the last follow-up (**Table 2**, **Supplementary Table S2**).

**Table 2:** Integrated chronological timeline.

Time	Clinical/organ involvement	Serum IgG4 (mg/L)	ESR/CRP	Key pituitary and endocrine findings	Pituitary MRI (stalk)	Treatment	Adherence	Outcome
Feb 2020	Painful right submandibular swelling (onset); initial diagnosis	—	—	—	—	MP 24 mg qd + LEF 20 mg qd (referring hospital); MP tapered to 4 mg qd	Treated locally	Improvement
Nov 2020	Referred to our center; IgG4-DS confirmed	6,840	—	—	—	MP 4 mg qd + LEF 20 mg qd	—	Remission
Jun 2021	Asymptomatic	—	—	—	—	Maintenance; regular follow-up at our center established (3–6 months)	Adherent	Remission
Sep 2021	—	—	—	—	—	All drugs self-discontinued	Interruption 1 (patient’s own decision)	—
Nov–Dec 2021	Bilateral submandibular and right lacrimal gland enlargement (relapse 1)	18,999 → 38,100 (peak)	16/1.64 (Dec 2021)	—	—	Prednisone 50 mg qd + CTX 150→100 mg qd; prednisone −5 mg/week	Restarted	Remission
Jan–May 2022	Asymptomatic	10,700 → 5,320 → 4,170	2–4/0.21–0.54	—	—	Prednisone 25 → 10 → 7.5 → 5 mg qd; iguratimod 25 mg bid (Mar; stopped Apr for gastric discomfort); MMF 1.0 g bid (May)	Adherent	Remission
Jul 2022	—	—	—	—	—	All drugs self-discontinued	Interruption 2	—
Sep 2022 (+2 months)	Asymptomatic	—	—	—	CT: small hyperdense suprasellar focus; MRI: no corresponding abnormality	Off treatment	Off treatment	Subclinical imaging change
Jan 2023 (+6 months)	Fatigue, cold intolerance, reduced libido, weight loss, polydipsia, nocturia	—	—	—	Sellar enlargement; 15 × 7 × 9 mm intrasellar mass with waist sign; 8 × 7 × 6 mm enhancing suprasellar lesion inseparable from stalk, optic chiasm displaced; stalk diameter not reported; abdominal imaging: RPF	No medication	Off treatment (-11 months, Jul 2022–Jun 2023)	IgG4-RH suspected
May 2023 (admission)	Partial anterior hypopituitarism and partial central DI; diagnosis established	19,407	20/1.55	PRL 10.9; FT4 0.82, TSH 2.461; ACTH 49.0, cortisol 11.6; GH 0.5, IGF-1 46 (low)	Stalk 4.0 mm	Admitted for treatment	—	Diagnosis
Jun–Sep 2023	Hypogonadism from Oct 2023	5,899 → 3,168 → 2,839 → 2,535 (nadir)	2–14/0.30–2.59	PRL 18.5 → 17.7 → 13.8 → 13.1; FSH 1.77 → 8.40 → 7.62; LH 0.29 → 2.83; T <0.1 → 0.36 → 0.58; GnRH test (23 Oct): LH 2.83 → 13.95 mIU/mL at 60 min	—	Prednisone 60 → 45 → 18.75 → 15 → 11.25 mg qd; CTX 100 mg qd; switched to MTX 20 mg qw with prednisone 11.25 mg qd (23 Oct 2023); testosterone 40 mg tid started	Adherent	Remission; gonadotroph reserve partially preserved
May–Sep 2024	Asymptomatic	—	—	PRL 10.7; FSH 7.54; LH 3.45; T 1.87 (May 2024)	Stalk 3.6 mm (Sep 2024)	Prednisone 7.5 mg qd (Jan 2024) → 5 mg qd (May 2024); MTX 20 mg qw	Adherent	Stable
Nov 2024	Clinically stable	6,807 (up from 2,535)	2/1.25 (normal)	PRL 11.8; FSH 4.63; LH 1.31; T 0.35; FT4 0.87, TSH 3.241; GH 0.1, IGF-1 62	Stable	Prednisone increased from 1.25 to 5 mg qd (26 Nov 2024); dose unchanged at the December 2024 visit; MTX 20 mg qw continued	Adherent; no interruption	Serological rise
Mar–Apr 2025	Clinical relapse	7,447	5/1.97	PRL 34.7; FSH 0.41, LH <0.2; FT4 0.73 (low), TSH 2.355; ACTH 30.6, cortisol 7.7; GH <0.05, IGF-1 54	Stalk 8.5 mm (Mar 2025, referring hospital)	MTX stopped → MMF 1.0 g bid (8 Apr); prednisone 5 mg qd continued; B-cell depletion recommended, declined (cost)	Adherent	Partial response
Nov 2025	Follow-up imaging at referring hospital	—	—	—	Stalk 7.0 mm	MMF 1.0 g bid; prednisone 5 mg qd; testosterone	Adherent	Improvement
Jan–Feb 2026 (last)	Clinically stable on hormone replacement and MMF	4,594	5/1.26	PRL 17.48; FSH 0.62, LH <0.2 (on testosterone); FT4 0.87, TSH 2.744 (normal); ACTH 19.4, cortisol 7.5; IGF-1 41 (low), GH 0.1; serum osmolality 294, urine osmolality 576; fluid intake >3 L/day	—	Prednisone 5 mg qd; MMF reduced to 0.75 g bid; testosterone 40 mg tid	Adherent	Persistent hypogonadism and partial central DI; thyrotroph and corticotroph function preserved

Visit-by-visit treatment doses and tapering schedules are listed in **Supplementary Table S1**. qd, once daily; bid, twice daily; qw, once weekly; tid, three times daily. CTX, cyclophosphamide; IGU, iguratimod; LEF, leflunomide; MMF, mycophenolate mofetil; MTX, methotrexate; RPF, retroperitoneal fibrosis; T, testosterone; PRL,prolactin; FT4, free T4; TSH, thyroid-stimulating hormone; ACTH, adrenocorticotropic hormone; IGF-1, insulin-like growth factor 1; LH, luteinizing hormone; ESR, erythrocyte sedimentation rate; CRP, C-reactive protein.

Notably, serum IgG4 remained elevated at 6,807 mg/L in November 2024 despite clinical stability and normal ESR and CRP levels. In this patient, re-elevation of serum IgG4 preceded subsequent clinical and radiological relapse. However, the interval between the November 2024 IgG4 rebound and the April 2025 clinical relapse was 5 months, and the exact onset time of IgG4 elevation cannot be pinpointed due to the sampling interval. Therefore, a direct causal link between the November 2024 IgG4 rise and subsequent tissue damage cannot be established. Although IgG4 elevation has been reported to precede clinical relapse by 2–6 months, consistent with progressive subclinical inflammation, a more rapid rise shortly before relapse cannot be excluded given interindividual variability in disease progression. Whether serial IgG4 measurement can reliably identify subclinical activity or predict relapse requires evaluation in larger, prospectively followed cohorts.

Because serum IgG4 may incompletely reflect disease activity (5, 24), we longitudinally assessed the other IgG subclasses. IgG1, IgG2, and IgG3 remained within reference ranges throughout, whereas total IgG and IgG4 increased to 31.68 g/L and 38,100 mg/L, respectively (**Supplementary Table S2**). Thus, the discordance between serum IgG4 and clinical activity was not associated with a shift toward IgG1, IgG2, or IgG3 predominance. Although IgG1/IgG3 subclass skewing may contribute to IgG4-disease activity discordance in other patients, our findings do not support this mechanism in the present case (5).

During the active phase following the first treatment interruption, serum IgG4 reached an exceptionally high level of 38,100 mg/L, with exacerbation of lacrimal and salivary gland involvement. Markedly elevated IgG4 concentrations have been associated with more extensive organ involvement and higher disease activity (25). Although severe polyclonal hypergammaglobulinaemia can rarely cause hyperviscosity syndrome (26), no clinical features of hyperviscosity developed.

### 4.4 Treatment exposure, adherence and interpretation of disease progression

Glucocorticoids remain the first-line therapy for inducing remission in IgG4-RD and IgG4-RH (7). In this patient, glucocorticoid therapy rapidly improved systemic inflammation, reduced serum IgG4 levels, and ameliorated pituitary MRI abnormalities. However, two patient-initiated treatment interruptions occurred in September 2021 and July 2022, both after symptom improvement led the patient to believe that the disease had resolved. Adherence was poor before the diagnosis of IgG4-related hypophysitis but improved substantially thereafter, with regular follow-up every 3–6 months.

Three distinct episodes of disease progression should be distinguished. The first relapse, in December 2021, occurred 3 months after the first treatment interruption. New pituitary involvement with retroperitoneal fibrosis and pituitary dysfunction developed between September 2022 and January 2023 after the second interruption, during which the patient remained off all immunosuppressive therapy for approximately 11 months. These two episodes therefore occurred in the context of treatment interruption rather than inadequate efficacy of ongoing immunosuppression. In contrast, radiological relapse was first detected on MRI in March 2025, and the third clinical relapse was confirmed in April 2025; this episode occurred despite continuous treatment without interruption. From November 2024, prednisone had been increased from 1.25 to 5 mg/day and was maintained with methotrexate 20 mg/week through April 2025 without documented deviations. This relapse occurred despite adherence during relatively low-intensity maintenance therapy, suggesting that prednisone 5 mg/day combined with methotrexate was insufficient to achieve sustained disease control in this patient.

We accordingly propose the following interpretation: In this patient, progression occurred in temporal association with documented treatment interruptions, while subsequent disease activity despite sequential csDMARD exposure suggested incomplete disease control. The relative contributions of non-adherence and pharmacological inadequacy cannot be determined from a single case.

### 4.5 B-cell depletion therapy: an individualized, not a generalized, indication

Increasing evidence indicates that B cells and plasmablasts play a central role in the pathogenesis of IgG4-RD (3, 4). Rituximab, an anti-CD20 monoclonal antibody, has shown efficacy in refractory or relapsing disease (27, 28), and the phase III MITIGATE trial demonstrated that inebilizumab significantly reduced relapse risk and increased glucocorticoid-free remission (29, 30). B-cell depletion therapy may be considered as an alternative treatment option for selected patients with high-risk critical organ involvement, or for those with persistent disease activity despite optimized conventional therapy at adequate doses and duration. Clinical decision-making should carefully balance infection risk, patient preference, and healthcare resource availability (31, 32).

The complex disease course observed in this case is confounded by multiple episodes of unplanned treatment interruption, and therefore cannot be used as direct evidence that conventional therapy is entirely ineffective. For patients with chronic HBV infection, B-cell depletion therapy carries a risk of HBV reactivation. Pre-treatment evaluation by an infectious disease specialist, baseline HBV viral load assessment, prophylactic antiviral therapy when indicated, and long-term virological monitoring are mandatory before initiating B-cell depletion therapy (33).

Other important considerations include treatment cost, drug accessibility, relapse risk after discontinuation, and the remaining uncertainty in the field regarding the optimal timing of initiation and the target population that derives maximum benefit. This single case cannot provide direct comparative evidence that earlier initiation of B-cell depletion therapy would have completely prevented the irreversible endocrine damage observed in this patient.

### 4.6 Chronic HBV infection as a possible contributing factor

Chronic HBV infection has been associated with extrahepatic immune-mediated manifestations, including vasculitis and glomerulonephritis (34). Proposed mechanisms include molecular mimicry, immune-complex deposition, and chronic antigen-driven B-cell/plasmablast activation with type I interferon signaling (34). Persistent HBV antigenic stimulation may have contributed to the markedly elevated IgG4 levels and relapsing disease course by sustaining B-cell/plasmablast activation, although a causal role for HBV in IgG4-RD remains unestablished. HBV infection also has important implications for B-cell depletion therapy, which carries a substantial risk of HBV reactivation. Guidelines recommend pre-treatment HBV screening and prophylaxis with a high-genetic-barrier antiviral initiated before or at the start of B-cell depletion and continued for 12–18 months thereafter, with serial HBV DNA and liver enzyme monitoring (33). The patient was already receiving tenofovir, with normal liver enzymes and stable virological markers, but specialist assessment and continued virological monitoring would remain necessary if B-cell depletion were initiated.

### 4.7 Limitations

This single-patient retrospective study has several limitations. Histopathological confirmation was unavailable, and treatment adherence before 2023 could not be objectively verified. Intermittent IgG4 measurements precluded precise determination of its rise before relapse, and total IgG was unavailable at the final assessment. Whole-body PET/CT, long-bone imaging, and cerebrospinal fluid analysis were not performed, so occult Erdheim-Chester disease involvement cannot be formally excluded. Differences in MRI scanners and incompletely retrievable acquisition parameters limited quantitative comparison of pituitary stalk measurements over time. GH provocative testing was not performed, and the long-term effects of B-cell depletion could not be assessed. The potential roles of IgG4 as an early relapse marker, the atypical enhancement pattern, and chronic HBV infection therefore remain hypothesis-generating.

## 5 Conclusion

This case provides a 5-year longitudinal clinical, serological, endocrine, and MRI evolution of organ-threatening IgG4-RD from IgG4-DS to IgG4-RH with hypogonadism. Its main value lies in the detailed longitudinal assessment of pituitary imaging, serological activity, and multi-axis endocrine function, which is rarely documented in this condition. Disease progression occurred mainly during treatment interruption, while residual activity persisted despite sequential conventional csDMARDs; however, given the single-patient nature of this report, associations between serum IgG4, treatment exposure, and relapse, as well as the potential benefit of earlier B-cell depletion, remain hypothesis-generating and require prospective validation.

## 6 Patient perspective

Written informed consent for participation and for publication of this case report, including any potentially identifiable images or data, was obtained from the patient himself. Ethical approval was not required for this study because it describes a single clinical case and did not involve experimental procedures, interventional research, or the collection of additional data beyond routine clinical care.

## Data Availability

The original contributions presented in the study are included in the article/**Supplementary Material**. Further inquiries can be directed to the corresponding authors.
