# Supplementary material for: Case report: Progressive organ-threatening IgG4-related disease despite conventional immunosuppression: lessons from a 5-year longitudinal follow-up
Source: Front Immunol. 2026 Oct 28;17:1975295. doi: 10.3389/fimmu.2026.1975295 (PMC13752170; doi:10.3389/fimmu.2026.1975295)
Supplement: Supplementary file 1 [file Table1.docx]

**Supplement Material**

## Supplementary Table S1. Complete visit-by-visit treatment history and adherence

| Visit / period | Glucocorticoid | Immunosuppressive therapy | Hormone replacement | Tapering or adjustment plan | Adherence and reason for change |
| --- | --- | --- | --- | --- | --- |
| Feb 2020 (referring hospital) | MP 24 mg qd | LEF 20 mg qd | — | MP tapered gradually to 4 mg qd | Initial treatment at the referring hospital |
| Jun 2021 | MP 4 mg qd | LEF 20 mg qd | — | Maintenance | Adherent; regular follow-up at our centre established (3–6 months) |
| Sep 2021 | — | — | — | — | Self-discontinued all medication (believed he had recovered) |
| Dec 2021 | Prednisone 50 mg qd | CTX 150 mg qd | — | Prednisone −5 mg qd per week after the first week; CTX reduced to 100 mg qd after 1 month | Restarted for relapse (IgG4 38,100 mg/L) |
| Jan 2022 | Prednisone 25 mg qd | CTX 100 mg qd | — | Prednisone −5 mg qd per week | Adherent |
| Mar 2022 | Prednisone 10 mg qd | CTX 100 mg qd plus iguratimod 25 mg bid | — | Prednisone −2.5 mg qd per month; CTX continued | Adherent |
| Apr 2022 | Prednisone 7.5 mg qd | CTX 100 mg qd; iguratimod stopped | — | — | Gastric discomfort attributed to iguratimod |
| May 2022 | Prednisone 5 mg qd | MMF 1.0 g bid | — | CTX replaced by MMF | Cumulative CTX dose reached |
| Jul 2022 | — | — | — | — | Second self-discontinuation (believed symptoms had resolved) |
| Jul 2022 – Jun 2023 | None | None | None | — | No immunosuppressive therapy for approximately 11 months |
| Jun 2023 | Prednisone 60 mg qd | CTX 100 mg qd | — | Prednisone −5 mg qd per week to approximately 20 mg qd, thereafter −1.25 mg qd per week | Treatment restarted at our centre (IgG4-RH diagnosed May 2023) |
| Jul 2023 | Prednisone 45 mg qd | CTX 100 mg qd | — | As above | Adherent |
| Aug 2023 | Prednisone 18.75 mg qd | CTX 100 mg qd | — | −5 mg qd per week to 20 mg qd, thereafter −1.25 mg qd per week | Adherent |
| Sep 2023 | Prednisone 15 mg qd | CTX 100 mg qd | — | −1.25 mg qd per week | Adherent |
| Oct 2023 | Prednisone 11.25 mg qd | MTX 20 mg qw | Testosterone 40 mg tid (started) | −1.25 mg qd per month; CTX replaced by MTX | Cumulative CTX dose reached; persistent hypogonadism |
| Jan 2024 | Prednisone 7.5 mg qd | MTX 20 mg qw | Testosterone 40 mg tid | −1.25 mg qd per month | Adherent |
| May 2024 | Prednisone 5 mg qd | MTX 20 mg qw | Testosterone 40 mg tid | −1.25 mg qd per month | Adherent |
| Nov 2024 | Prednisone 1.25 mg qd, increased to 5 mg qd on 26 Nov 2024 | MTX 20 mg qw | Testosterone 40 mg tid | Prednisone increased to 5 mg qd | Serum IgG4 rose to 6,807 mg/L with stable clinical and imaging findings; no interruption |
| Dec 2024 | Prednisone 5 mg qd | MTX 20 mg qw | Testosterone 40 mg tid | Regimen unchanged | Adherent |
| Nov 2025 (referring hospital) | Prednisone 5 mg qd | MMF 1.0 g bid | Testosterone 40 mg tid | No treatment change documented | Follow-up imaging only (stalk 7.0 mm) |
| Apr 2025 | Prednisone 5 mg qd | MTX stopped, replaced by MMF 1.0 g bid | Testosterone 40 mg tid | MTX replaced by MMF | Relapse (stalk 8.5 mm); B-cell depletion therapy recommended but declined for financial reasons |
| Feb 2026 (last visit) | Prednisone 5 mg qd | MMF 1.0 g bid, reduced to 0.75 g bid | Testosterone 40 mg tid | MMF reduced to 0.75 g bid | Clinical improvement (stalk 7.0 mm) |

Notes: qd, once daily; bid, twice daily; qw, once weekly; tid, three times daily. MP, methylprednisolone; LEF, leflunomide; CTX, cyclophosphamide; MMF, mycophenolate mofetil; MTX, methotrexate. Prednisone was administered as prednisone acetate. Doses shown are those documented at the corresponding visit; the tapering plan shown is that recorded in the clinical notes at each visit. The cumulative cyclophosphamide dose was monitored prospectively and the drug was replaced once the cumulative limit was reached. No adverse event other than iguratimod-related gastric discomfort was documented.

## Supplementary Table S2. Serial serum IgG4, total IgG, IgG subclasses and inflammatory markers

| Date | IgG4 (mg/L) | Total IgG (g/L) | IgG1 (mg/L) | IgG2 (mg/L) | IgG3 (mg/L) | ESR (mm/h) | CRP (mg/L) | LDH (U/L) |
| --- | --- | --- | --- | --- | --- | --- | --- | --- |
| Nov 2020 | 6,840 | — | — | — | — | — | — | — |
| Nov 2021 | 18,999 | — | — | — | — | — | — | — |
| Dec 2021 | 38,100 | 31.68 | 9,380 | 2,010 | 419 | 16 | 1.64 | — |
| Jan 2022 | 10,700 | 10.88 | 4,850 | 3,110 | 130 | 2 | 0.21 | — |
| Feb 2022 | 5,320 | 9.92 | 5,240 | 2,710 | 199 | 4 | 0.54 | — |
| May 2022 | 4,170 | — | — | — | — | — | — | — |
| May 2023 | 19,407 | 21.10 | 6,574 | 2,079 | 366 | 20 | 1.55 | 215 |
| Jun 2023 | 5,899 | 11.38 | 4,092 | 1,373 | 177 | 2 | 0.30 | 255 |
| Aug 2023 | 3,168 | 8.38 | 4,210 | 1,278 | 218 | 10 | 2.59 | 361 |
| Sep 2023 | 2,839 | 8.34 | 4,865 | 1,426 | 216 | 14 | 0.99 | 348 |
| Oct 2023 | 2,535 | 8.51 | 5,374 | 1,705 | 224 | 10 | 1.00 | 322 |
| Jan 2024 | — | — | — | — | — | — | — | 305 |
| May 2024 | — | — | — | — | — | — | — | 270 |
| Nov 2024 | 6,807 | 12.16 | 5,978 | 2,266 | 185 | 2 | 1.25 | 256 |
| Apr 2025 | 7,447 | 12.40 | 5,441 | 1,841 | 199 | 5 | 1.97 | 229 |
| Jan 2026 | 4,594 | not done | 4,917 | 2,076 | 208 | 5 | 1.26 | 214 |

Reference ranges: IgG4 80–1,400 mg/L; total IgG 7.00–17.00 g/L; IgG1 4,900–11,400 mg/L; IgG2 1,500–6,400 mg/L; IgG3 80–1,400 mg/L; ESR 0–15 mm/h; CRP <3.00 mg/L; LDH 0–250 U/L. A dash indicates that the analyte was not measured. Note that IgG1, IgG2 and IgG3 remained within their reference ranges at every time point, whereas total IgG and IgG4 were markedly elevated, indicating that the discordance between serum IgG4 and clinical activity in this patient was not attributable to a shift towards another IgG subclass.

## Supplementary Table S3. Serial endocrine data and water-balance indices

| Date | PRL (ng/mL) | FSH (IU/L) | LH (IU/L) | Testosterone (ng/mL) | FT3 (pg/mL) | FT4 (ng/dL) | TSH (µIU/mL) | ACTH (pg/mL) | Cortisol (µg/dL) | GH (ng/mL) | IGF-1 (ng/mL) | Serum osmolality (mOsm/kg) | Urine osmolality (mOsm/kg) |
| --- | --- | --- | --- | --- | --- | --- | --- | --- | --- | --- | --- | --- | --- |
| May 2023 | 10.9 | — | — | — | 2.94 | 0.82 | 2.461 | 49.0 | 11.6 | 0.5 | 46 | — | — |
| Jun 2023 | 18.5 | 1.77 | 0.29 | <0.1 | — | — | — | — | — | — | — | — | — |
| Aug 2023 | 17.7 | — | — | — | — | — | — | — | — | — | — | — | — |
| Sep 2023 | 13.8 | 8.40 | — | 0.36 | — | — | — | 22.7 | 11.9 | — | 132 | — | — |
| Oct 2023 | 13.1 | 7.62 | 2.83 | 0.58 | — | — | — | — | — | — | — | — | — |
| Jan 2024 | 14.0 | 8.75 | 2.89 | 1.91 | 3.46 | 0.96 | 3.037 | — | — | 0.4 | 97 | 296 | 683 |
| May 2024 | 10.7 | 7.54 | 3.45 | 1.87 | 3.64 | 0.92 | 1.838 | — | — | 0.1 | 110 | — | — |
| Nov 2024 | 11.8 | 4.63 | 1.31 | 0.35 | 3.95 | 0.87 | 3.241 | — | — | 0.1 | 62 | — | — |
| Apr 2025 | 34.7 | 0.41 | <0.2 | 1.63 | 3.95 | 0.73 | 2.355 | 30.6 | 7.7 | <0.05 | 54 | — | — |
| Jan 2026 | 17.48 | 0.62 | <0.2 | 0.29 | 3.06 | 0.87 | 2.744 | 19.4 | 7.5 | 0.1 | 41 | 294 | 576 |

Reference ranges: PRL 2.6–13.1 ng/mL; FSH 1.27–19.26 IU/L; LH 1.24–8.62 IU/L; testosterone 1.75–7.81 ng/mL; FT3 1.80–4.10 pg/mL; FT4 0.81–1.89 ng/dL; TSH 0.380–4.340 µIU/mL; ACTH 7.2–63.3 pg/mL; cortisol 4.0–22.3 µg/dL; GH <2.0 ng/mL; IGF-1 53–215 ng/mL; serum osmolality 275–305 mOsm/kg; urine osmolality 40–1,400 mOsm/kg. A dash indicates that the analyte was not measured. All testosterone samplings were performed after the patient had paused testosterone replacement therapy for a full week, to completely eliminate the interference of exogenous supplementary drugs on the results of serum testosterone detection. Water balance: a water-deprivation test was not performed and 24-h urine volume was not routinely measured; the patient reported a daily fluid intake exceeding 3 L. Serum osmolality remained within the reference range with urine osmolality of 576–683 mOsm/kg, consistent with partial central diabetes insipidus.

## Supplementary Table S4. Pituitary MRI findings, sequences and imaging planes

| Date | Maximum pituitary stalk diameter | Panel | Sequence and imaging plane | Additional findings |
| --- | --- | --- | --- | --- |
| Sep 2022 | not measured | — | Non-contrast cranial CT and pituitary MRI | Small hyperdense focus in the suprasellar cistern on CT; no corresponding abnormality on MRI |
| Jan 2023 | not reported | — | Contrast-enhanced pituitary MRI | Sellar enlargement with a 15 × 7 × 9 mm intrasellar mass exhibiting a waist sign; 8 × 7 × 6 mm enhancing suprasellar lesion inseparable from the pituitary stalk, with upward displacement of the optic chiasm; retroperitoneal fibrosis on abdominal imaging |
| May 2023 | 4.0 mm | a | OSag fs T1 FSE+, fat-saturated CE-T1WI, mid-sagittal sellar plane | — |
| May 2023 |  | b | OCor T1 FSE+, CE-T1WI, mid-pituitary stalk coronal plane | — |
| May 2023 |  | c | OCor T2 FRFSE, T2WI, mid-pituitary body coronal plane | — |
| Sep 2024 | 3.6 mm | d | eBTFE_BH SENSE, CE-T1WI, mid-sagittal plane | Stable findings during maintenance therapy |
| Sep 2024 |  | e | T1W_TSE_Dyn(C+), DCE-T1WI, mid-stalk coronal plane | — |
| Mar 2025 | 8.5 mm | f | eT1W_TSE, CE-T1WI, mid-pituitary stalk coronal plane | Re-enlargement of nodular stalk thickening (relapse) |
| Mar 2025 |  | g | sT1W_TSE_Dyn(C+), DCE-T1WI, mid-pituitary body coronal plane | — |
| Mar 2025 |  | h | eT1W_TSE, CE-T1WI, mid-sagittal sellar plane | — |
| Nov 2025 | 7.0 mm | i | CE-T1WI (eT1W_TSE), mid-sagittal plane | Partial regression after switching to mycophenolate mofetil |
| Nov 2025 |  | j | DCE-T1WI (T1W_TSE_Dyn), mid-stalk coronal plane | — |

Notes: Maximum pituitary stalk diameter was defined as the maximum diameter of the stalk on contrast-enhanced T1-weighted images in the mid-sagittal sellar plane and the mid-stalk coronal plane, and was recorded as reported in the clinical radiology reports; no independent blinded re-measurement was performed. Gadopentetate dimeglumine (Gd-DTPA) was used as the intravenous contrast agent in all contrast-enhanced examinations. The magnetic field strength, slice thickness and the administered contrast dose were not uniformly recorded and could not be retrieved retrospectively; examinations were performed on different scanners at two institutions, so small differences in stalk diameter between time points should be interpreted with caution. The January 2023 examination did not report a stalk diameter and is therefore not included in the serial measurement series. CE-T1WI, contrast-enhanced T1-weighted imaging; DCE-T1WI, dynamic contrast-enhanced T1-weighted imaging; T2WI, T2-weighted imaging; fs, fat-saturated; OSag, oblique sagittal; OCor, oblique coronal.

## Supplementary Table S5. CARE checklist

| Number | CARE checklist item | Location in the revised manuscript |
| --- | --- | --- |
| 1 | Title: diagnosis or intervention of interest, plus the words "case report" | Title |
| 2 | Key words: two to five key words including "case report" | Keywords |
| 3a | Abstract: introduction — what is unique and what it adds | Abstract, Objective |
| 3b | Abstract: main symptoms, clinical findings, main diagnosis, interventions and outcomes | Abstract, Methods and Results |
| 3c | Abstract: conclusion, including the main take-away lessons | Abstract, Conclusions |
| 4a | Introduction: background — summary of the relevant literature | Section 1, paragraphs 1–3 |
| 4b | Introduction: rationale for reporting the case | Section 1, paragraph 4 |
| 5a | Patient information: de-identified demographic and other specific information | Section 2.1 |
| 5b | Patient information: main symptoms of the patient | Sections 2.1–2.3 |
| 5c | Patient information: medical, family and psychosocial history, including relevant genetic information | Section 2.1 (chronic HBV infection, adherence history, financial constraints affecting treatment choice) |
| 5d | Patient information: relevant past interventions and their outcomes | Sections 2.1–2.2; Supplementary Table S1 |
| 6 | Clinical findings: description of the relevant physical examination and other clinical findings | Sections 3; Table 1 |
| 7 | Timeline: historical and current information from the episode of care | Table 1; Figure 1; Supplementary Table S1 |
| 8a | Diagnostic assessment: diagnostic testing and results | Sections 2.4, 4.4; Supplementary Tables S2–S4 |
| 8b | Diagnostic assessment: diagnostic challenges | Sections 2.4, 4.4; Section 4.1 |
| 8c | Diagnostic assessment: diagnostic reasoning, including differential diagnoses | Section 4.2; Table 2 |
| 8d | Diagnostic assessment: prognostic characteristics where applicable | Section 4.1 |
| 9a | Therapeutic intervention: types of intervention | Sections 2.1–2.5; Table 1; Supplementary Table S1 |
| 9b | Therapeutic intervention: administration of intervention, including dose, strength and duration | Table 1; Supplementary Table S1 |
| 9c | Therapeutic intervention: changes in intervention with explanations | Table 1; Supplementary Table S1; Section 4.4 |
| 10a | Follow-up and outcomes: clinician-assessed and patient-assessed outcomes | Sections 2.5–2.6; Table 1 |
| 10b | Follow-up and outcomes: important follow-up diagnostic and other test results | Section 2.6; Supplementary Tables S2–S4 |
| 10c | Follow-up and outcomes: intervention adherence and tolerability | Sections 2.5–2.7; Section 4.5; Supplementary Table S1 |
| 10d | Follow-up and outcomes: adverse and unanticipated events | Section 2.7 |
| 11a | Discussion: strengths and limitations of the management of this case | Section 4.7 |
| 11b | Discussion: relevant medical literature | Sections 4.1–4.7 |
| 11c | Discussion: rationale for conclusions, including an assessment of cause and effect | Sections 4.4–4.7; Section 5 |
| 11d | Discussion: main take-away lessons | Section 5 |
| 12 | Patient perspective: the patient's perspective on the care received | Section 6 |
| 13 | Informed consent: whether the patient gave informed consent for publication | Section 6 |

**Supplement Figure S1. Longitudinal Changes in Hypothalamic-Pituitary-Gonadal Axis Hormone Levels at Key Clinical Time Points**

The gonadal axis was the most severely affected, with irreversible functional impairment. Prolactin(PRL) levels remained persistently elevated, whereas follicle-stimulating hormone(FSH) and luteinizing hormone(LH) progressively declined, resulting in irreversible hypogonadotropic hypogonadism. Endogenous testosterone remained markedly reduced, normalizing only transiently during replacement therapy and rapidly declining after treatment withdrawal.

**Supplement Figure S2. Longitudinal Changes in Hypothalamic-Pituitary-Thyroid axis Hormone Levels at Key Clinical Time Points**

Thyroid axis function remained largely preserved. Thyroid-stimulating hormone(TSH) and free triiodothyronine(FT3) remained within the reference range throughout follow-up, with only a transient mild reduction in free thyroxine(FT4) in April 2025, indicating minimal thyroid axis involvement.

**Supplement Figure S3. Longitudinal Changes in Hypothalamic-Pituitary-Adrenal Axis Hormone Levels at Key Clinical Time Points**

Adrenal axis function remained largely preserved. Adrenal axis hormones remained within the reference range, although total serum cortisol gradually declined during glucocorticoid tapering.

**Supplement Figure S4. Longitudinal Changes in Hypothalamic-Pituitary-Growth Hormone Axis Hormone Levels at Key Clinical Time Points**

The Growth Hormone(GH) axis was moderately affected, despite normal basal GH levels, repeatedly decreased insulin-like growth factor-1(IGF-1) levels suggested probable GH axis involvement. However, adult GH deficiency could not be confirmed because the insulin tolerance test was not performed, and chronic fibroinflammation together with prolonged glucocorticoid therapy may also have contributed to reduced IGF-1 levels.
