# Supplementary material for: Case report: Progressive organ-threatening IgG4-related disease despite conventional immunosuppression: lessons from a 5-year longitudinal follow-up
Source: Front Immunol. 2026 Oct 28;17:1975295. doi: 10.3389/fimmu.2026.1975295 (PMC13752170; doi:10.3389/fimmu.2026.1975295)
Supplement: Supplementary file 2 [file Table2.docx]

## Supplementary Table S5. CARE checklist

| Number | CARE checklist item | Location in the revised manuscript |
| --- | --- | --- |
| 1 | Title: diagnosis or intervention of interest, plus the words "case report" | Title |
| 2 | Key words: two to five key words including "case report" | Keywords |
| 3a | Abstract: introduction — what is unique and what it adds | Abstract, Objective |
| 3b | Abstract: main symptoms, clinical findings, main diagnosis, interventions and outcomes | Abstract, Methods and Results |
| 3c | Abstract: conclusion, including the main take-away lessons | Abstract, Conclusions |
| 4a | Introduction: background — summary of the relevant literature | Section 1, paragraphs 1–3 |
| 4b | Introduction: rationale for reporting the case | Section 1, paragraph 4 |
| 5a | Patient information: de-identified demographic and other specific information | Section 2.1 |
| 5b | Patient information: main symptoms of the patient | Sections 2.1–2.3 |
| 5c | Patient information: medical, family and psychosocial history, including relevant genetic information | Section 2.1 (chronic HBV infection, adherence history, financial constraints affecting treatment choice) |
| 5d | Patient information: relevant past interventions and their outcomes | Sections 2.1–2.2; Supplementary Table S1 |
| 6 | Clinical findings: description of the relevant physical examination and other clinical findings | Sections 3; Table 1 |
| 7 | Timeline: historical and current information from the episode of care | Table 1; Figure 1; Supplementary Table S1 |
| 8a | Diagnostic assessment: diagnostic testing and results | Sections 2.4, 4.4; Supplementary Tables S2–S4 |
| 8b | Diagnostic assessment: diagnostic challenges | Sections 2.4, 4.4; Section 4.1 |
| 8c | Diagnostic assessment: diagnostic reasoning, including differential diagnoses | Section 4.2; Table 2 |
| 8d | Diagnostic assessment: prognostic characteristics where applicable | Section 4.1 |
| 9a | Therapeutic intervention: types of intervention | Sections 2.1–2.5; Table 1; Supplementary Table S1 |
| 9b | Therapeutic intervention: administration of intervention, including dose, strength and duration | Table 1; Supplementary Table S1 |
| 9c | Therapeutic intervention: changes in intervention with explanations | Table 1; Supplementary Table S1; Section 4.4 |
| 10a | Follow-up and outcomes: clinician-assessed and patient-assessed outcomes | Sections 2.5–2.6; Table 1 |
| 10b | Follow-up and outcomes: important follow-up diagnostic and other test results | Section 2.6; Supplementary Tables S2–S4 |
| 10c | Follow-up and outcomes: intervention adherence and tolerability | Sections 2.5–2.7; Section 4.5; Supplementary Table S1 |
| 10d | Follow-up and outcomes: adverse and unanticipated events | Section 2.7 |
| 11a | Discussion: strengths and limitations of the management of this case | Section 4.7 |
| 11b | Discussion: relevant medical literature | Sections 4.1–4.7 |
| 11c | Discussion: rationale for conclusions, including an assessment of cause and effect | Sections 4.4–4.7; Section 5 |
| 11d | Discussion: main take-away lessons | Section 5 |
| 12 | Patient perspective: the patient's perspective on the care received | Section 6 |
| 13 | Informed consent: whether the patient gave informed consent for publication | Section 6 |
